# Patient-Surgeon Communication in Thoracic Surgery: Insights From a European Multi-Country Survey on the Perioperative Experience

**DOI:** 10.1093/icvts/ivaf228

**Published:** 2025-09-25

**Authors:** Jonathan Pineda, Obieda Atiyani, Nicolas Moreno, Nuria Novoa, Sergio Bolufer Nadal, Peter Zsoldos, Karel Pfeuty, Mahmoud Ismail, Peter Licht, Mario Nosotti, Majed Refai, Elizabeth Belcher, Melanie Jenkins, Marcin Zielinski, Róbert Baláž, Ivan Kuhajda, Debra Montague, Miro Janik, József Furák, Ann-Marie Baird, Gianluca Casali, Cecilia Pompili

**Affiliations:** Department of General Surgery, Rutgers University NJMS, 185 S Orange Ave, Newark, NJ 07103, United States; Department of General Surgery, University of Cincinnati, 2600 Clifton Ave, Cincinnati, OH 45221, United States; Department of Thoracic Surgery, Hospital Universitario Ramón y Cajal, M-607, Km.9, 100, Fuencarral-El Pardo, Madrid 28034, Spain; Department of Thoracic Surgery, Hospital Universitario Puerta de Hierro Majadahonda, C. Joaquin Rodrigo, 1, Majadahonda, Madrid 28222, Spain; Department of Thoracic Surgery, Hospital General Universitario Dr. Balmis de Alicante, Av. Pintor Baeza, 12, Alicante, 03010, Spain; Department of Thoracic Surgery, Aladár Petz County Teaching Hospital, Vasvári Pál u. 2 - 4Györ9024, Hungary; Department of Thoracic and Vascular Surgery, Centre Hospitalier de Saint Brieuc, 10 Rue Marcel Proust, Saint-Brieuc 22000, France; Department of Thoracic Surgery, Klinikum Ernst von Bergmann, Academic Hospital of the Charité, Universitätsmedizin Berlin, Charlottenstrabe 72, Postdam 14467, Germany; Department of Cardiothoracic Surgery, Odense University Hospital, J.B. Winsløws Vej 4, Odense 5000, Denmark; Department of Thoracic Surgery, Policlinico of Milan, Via Francesco Sforza, 35, Milan 20122, Italy; Department of General Thoracic Surgery, Ospedali Riuniti di Ancona, Via Conca, 71, Ancona 60126, Italy; Department of Thoracic Surgery, John Radcliffe Hospital, Oxford University Hospitals, Headley Way, Headington, Oxford OX3 9DU, United Kingdom; Department of Thoracic Surgery, St. George’s Hospital NHS Foundation Trust, Blackshaw Rd, London SW17 OQT, United Kingdom; Department of Thoracic Surgery, The Zakopane Pulmonary Hospital, Gladkie 1, Zakopane 34-500, Poland; Department of Thoracic Surgery, University Hospital Bratislava, Mickiewiczova 2247, Bratislava 811 07, Slovakia; Department of Thoracic Surgery, Institute for Pulmonary Diseases of Vojvodina, University of Novi Sad, Institutski put 4, Sremska Kamenic 21204, Serbia; Lung Cancer Europe Organization (LuCE), Effingerstrasse 30, Bern 3012, Switzerland; Department of Thoracic Surgery, University Hospital Bratislava, Mickiewiczova 2247, Bratislava 811 07, Slovakia; Department of Thoracic Surgery, University of Szeged, Dugonics tér 13, Szeged 6720, Hungary; Lung Cancer Europe Organization (LuCE), Effingerstrasse 30, Bern 3012, Switzerland; Johnson and Johnson MedTech, Medical Affairs, 1000N US-202, Raritan, NJ 08869, United States; Department of Thoracic Surgery, Institute for Clinical and Applied Health Research, University of Hull, Cottingham Road, Hull HU6 7RX, United Kingdom

**Keywords:** patient-related outcomes measures, enhanced recovery after surgery, thoracic surgery, survey, patient-surgeon communication

## Abstract

**Objectives:**

Many advancements have occurred in surgery from the technical side with increasingly sophisticated minimally invasive surgical options to the patient care side with the advent of ERAS and patient-related outcomes research. Patient-related outcomes research has allowed providers to focus on what is most important to a patient when it comes to quality of life; however, an ill-defined disconnect persists between the desires of patients and the perspectives of surgeons on what the patient values the most.

**Methods:**

A rigorously designed multi-country European survey of 9 carefully curated questions meant to mimic the perioperative journey for both the patient and the surgeon distributed through an online link or paper version who recently underwent thoracic surgery and to surgeons involved with thoracic surgical care to explore the possible disconnect of perceptions of care throughout the perioperative journey.

**Results:**

A total of 444 participants (230 patients and 214 surgeons) from different parts of Europe responded to the survey. Noted discrepancies were found throughout the preoperative, intraoperative, and postoperative phases when it came to perception of information given and understood, what was communicated, and how care was implemented.

**Conclusions:**

This study identifies critical gaps in the communication and perception of surgical care between patients and surgeons, emphasizing the need for the implementation of shared decision-making and increasing awareness of enhanced holistic support throughout the perioperative journey.

## Graphical abstract

**Figure:**
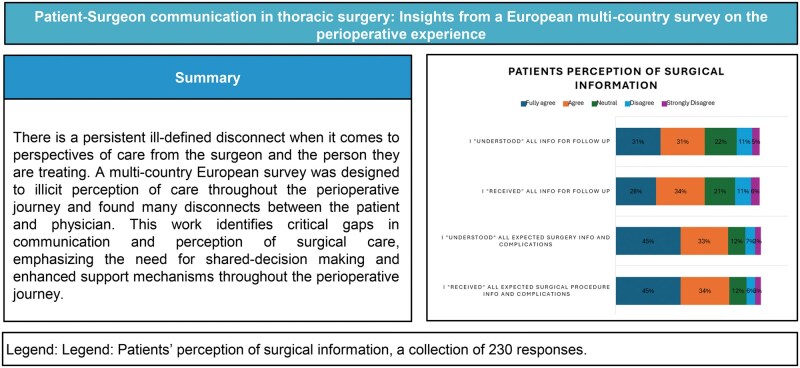

## INTRODUCTION

In the past couple of decades, thoracic surgery has experienced a number of key advancements in the field. From a technical aspect, minimally invasive surgery has rapidly been adopted with the video-assisted thoracic surgery (VATS) technique and robotic surgery. With the advantages of no rib spreading and equal oncologic success as open thoracotomy, VATS has resulted in fewer complications, improved quality of life, shorter length of stay, and even superior compliance with adjuvant therapies due to the reduced decline of functional status compared to open surgery.^1–3^

Enhanced recovery after surgery (ERAS) has been established to optimize the patient during and after surgery with a particular holistic approach including rehabilitation around surgery aiming at reducing surgical complications and length of stay.^4^ The initial focus of ERAS was primarily on clinical outcomes resulting from ERAS interventions, as interpreted by physicians, without actively assessing the quality of life experienced by the patients. However, a strong trend towards studying disease processes in relation to patient-related outcomes, otherwise known as patient-reported outcome measures (PROMs), has emerged.^5^ PROMS are used to capture the impact of an illness or health condition directly from the patient, without interpretation by a clinician or caregiver. So far, research has indicated that PROMs can help detect, manage, and monitor problems, while also facilitating patient-doctor communication.^6^^,^^7^ Despite knowing PROMS can help drive treatment course and can impact patients’ long-term satisfaction and wellbeing, a recent survey by the European Society of Thoracic Surgeons (ESTS) found that more than half of respondents were not collecting quality of life metrics.^8^ How can we advocate for people with lung cancer if we do not assess their preferences and potential impact on their daily lifestyle? There seems to be a disconnect between clinicians and patients in what actually matters to patients in their care.^9^ In thoracic surgery, there has been evidence that shows that patients actually want a more collaborative role in their care than they actually have at present.^10^^,^^11^ These factors underscore the disconnect that can exist between the patient and the surgeon in their communication. So far, there is no literature specifically in thoracic surgery assessing patients’ perception of their surgical journey.

By systematically analysing the perioperative journey from both patient and surgeon perspectives, this study aims to identify communication gaps and unmet needs, ultimately to inform the fostering of a more person-centred approach in thoracic surgery.

## METHODS

### Ethical statement

Ethical approval was not sought as this project was deemed a service evaluation, and no personally identifiable data were collected in accordance with institutional guidelines. Formal written consent was obtained from all participants before starting the questionnaire.

### Study design and population

This cross-sectional survey was conducted across various European countries, focusing on thoracic surgeons and the patients they are treating for lung cancer. The survey for patients was distributed by Lung Cancer Europe (LuCE), the patient association through LinkedIn, Facebook, and WhatsApp groups.

The inclusion criteria were for individuals who had undergone thoracic surgery, ensuring relevant and current insights into their postoperative experiences with no particular time-frame limit to increase response rates. Simultaneously, general thoracic surgeons from multiple European countries were invited to participate by email or the national surgical community.

### Survey design

An online questionnaire was developed starting from a literature review, and through a process involving researchers and clinicians included in the steering committee which included thoracic surgeons, patient representatives and experts with a diverse geographical distribution.

The questionnaire was pilot-tested by 3 researchers, including patient advocates not involved in the project, who were invited to evaluate the completeness of the questionnaire, the comprehensibility of the questions and the answer options, to identify relevant aspects not considered, and the time needed to complete the questionnaire. Some questions were re-formulated, and layout changes were made on the basis of these suggestions, especially limiting the use of medical jargon.

The final version of the survey (**Supplementary Appendix**) was constructed to reflect the journey of patients undergoing thoracic surgery through the entire treatment continuum. Nine questions in total were given to both surgeons and patients who followed the main aspects of a perioperative journey that can be applicable in all the countries involved. Questions were formulated to elicit specific insights within a specific domain of each phase of care.

- *Preoperative decision-making and planning*: Patients and surgeons were asked about the level of support in planning, for surgery and clarity in goals of surgery, recovery and complications.
- *In-hospital experience*: Feedback was gathered from patients and surgeons about the care provided during the hospital stay, including staff communication, pain management, and overall satisfaction with the surgical experience.
- *Postoperative care and recovery*: Participants provided data on their experiences during recovery, including their interface with healthcare services following discharge and the adequacy of postoperative support and planning.

By incorporating this structured approach, the survey aimed to highlight not only the clinical aspects of care but also the emotional and psychological dimensions experienced by patients throughout their surgical journey. The questions were asked with a multitude of possible answers, similar to a Likert scale, in order to elicit a better understanding of the patient experience. For example, when asked the patient if they “understood” all the information for surgery, instead of just “yes or no,” we gave the option of “Fully understood” and “Understood” or “disagree” or “strongly disagree.” Each question was given to all participants and therefore formed our percentages. Each question was given to all participants and therefore formed our percentages. Survey data were collected and managed using SurveyMonkey software (SurveyMonkey Inc., San Mateo, CA, USA).

## RESULTS

**Figure 1.**
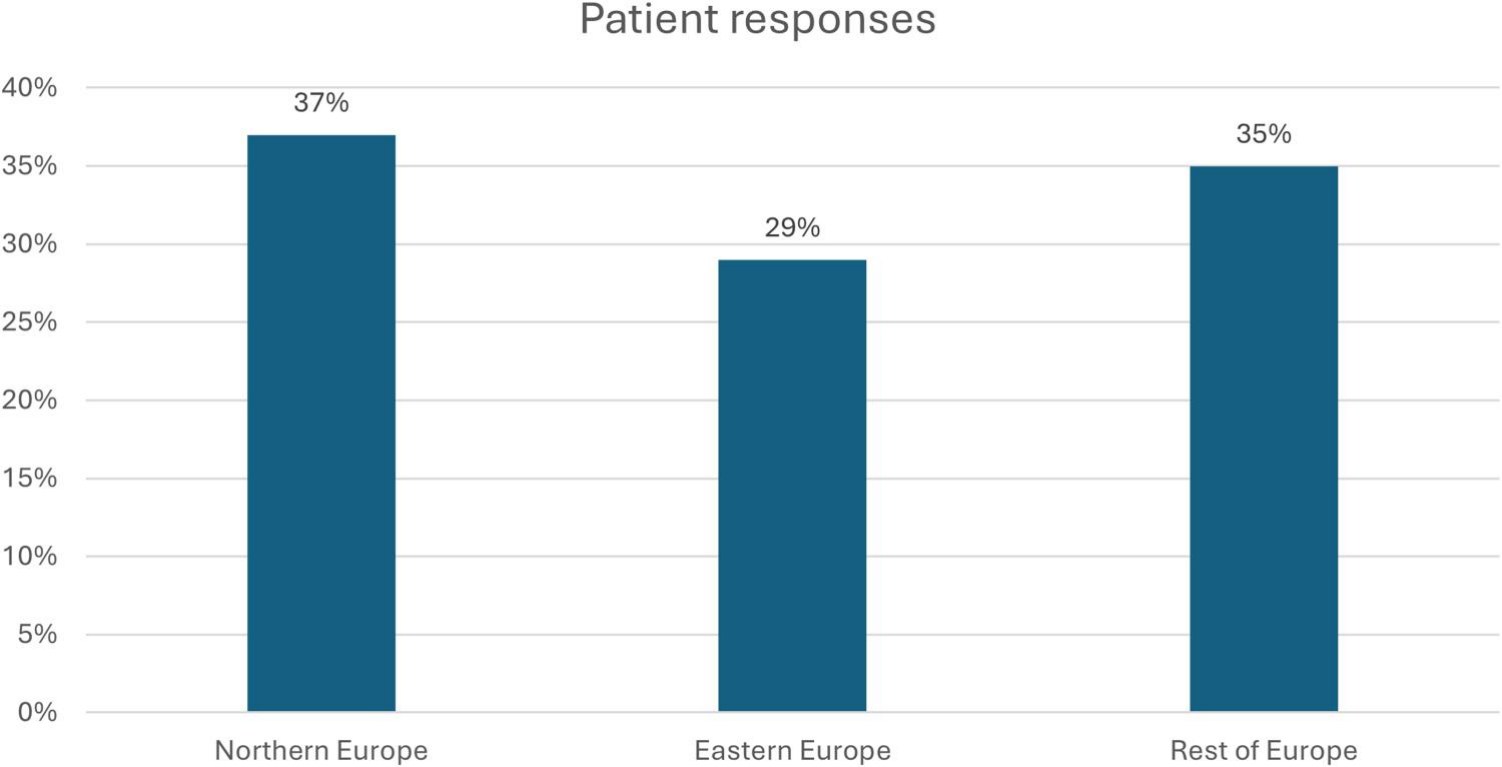
Patient Survey Results by European Region Based on Primary Language Spoken. Total of 230 responses

**Figure 2.**
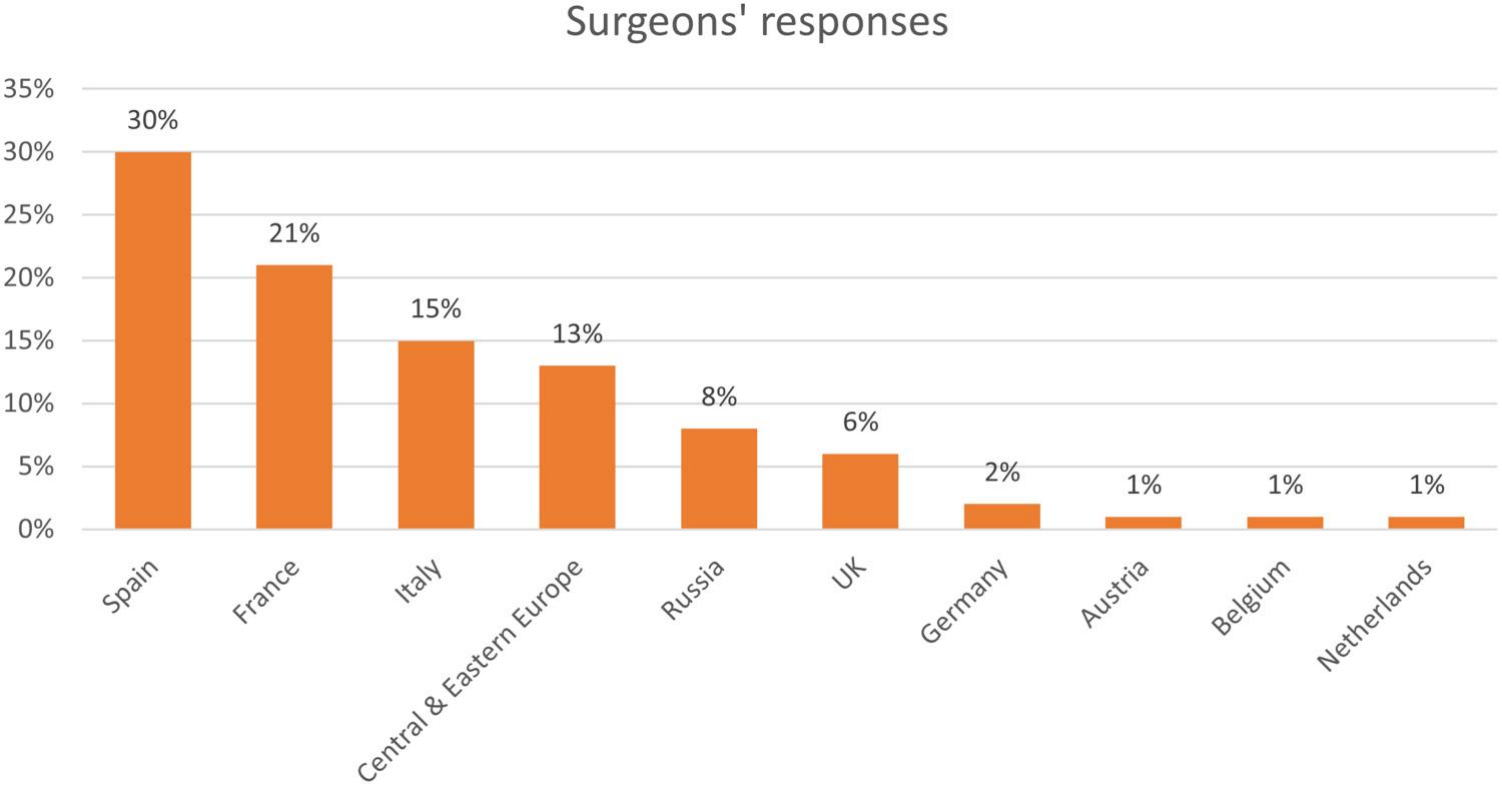
Surgeon Survey Results by Country out of 214 Surgeon Responses

The surgeons’ survey was open from April 2024 until May 2024. The patient surveys had 2 waves, 1 that opened from April 2024 until May 2024 that came back with limited participation, so a second wave of the survey was distributed that opened from November 2024 until December 2024. It was distributed throughout all regions of Europe (**Figures 1 and 2**). The survey was socialized via LuCE channels. LuCE is a European umbrella advocacy organization, which represents people living with or at risk of lung cancer. Due to the General Data Protection Regulation, we could not record the specific country of origin for the patient survey responses. In order to attempt any geographical categorization of responses, we assumed the patient would answer in their native language. Also, we did not collect a specific response rate due to how it was socialized.

### Participant demographics

A total of 230 patients and 214 surgeons completed the questionnaires. Patient respondents were spread through Europe, relatively evenly with 37% from Northern Europe, 29% from Eastern Europe, and 35% from the rest of Europe. While most surgeon respondents were from Spain (30%), France (21%), Italy (15%), and 13% from Central and Eastern Europe. The survey was created in multiple different languages including German, English, Hungarian, Slovakian, Italian, Spanish, and Polish.

### Surgeons’ survey

From the survey, we found that around 64% of surgeons stated they have specialized pulmonary nurses, while the majority (80%) stated they have been successful in adopting ERAS into their department (**Table 1**). The most common postoperative complications that were communicated were prolonged air leak (92%), bleeding (89%), and pain (86%) with even less percentage of discussion when it came to infections (77%) and even more in mortality (61%). We found that most of the time, surgeons communicate with the patient/family after surgery, but about only one-third of these conversations are done by both the surgeon and a nurse. Most of the time, surgeons felt like the surgery went as expected or better, and none of them thought it went worse than expected. The majority of surgeons (74%) either saw their patients up to 3 months (43%) or over 12 months (31%) postoperative. Surgeons referred their patients to a radiation/oncologist (79%) with only half responding that they sent their patient to a respiratory therapist (52%) or a general practitioner (41%). Only about a third of surgeons have fully adopted some version of remote follow-up, which when utilized is typically by telephone (74%).

**Table 1.** Surgeon Survey

Surgeon’s survey	Total number	%
**Do you have specialized pulmonary nurses?**		
Yes	137	64%
No	77	36%
**How successful have you been at adopting ERAS in your department?**		
Very successful	65	30%
Successful	108	50%
Minimally successful	30	14%
Not at all successful	11	5%
**Pre-operatively, which possible complications are fully communicated to patients?**		
Prolonged air leak	197	92%
Bleeding	191	89%
Pain	184	86%
Infections	165	77%
Mortality	131	61%
Respiratory failure	116	54%
Pulmonary embolism	94	44%
**Who communicates with the patient/family after the surgery?**		
Surgeon	133	62%
Both	77	36%
Other	2	1%
Nurse	2	1%
**On average, how do you think your patients feel the surgery went?**		
Better than expected	91	42%
As expected	123	57%
Worse than expected	0	0%
**On average, how long do you see your patients in clinic after discharge?**		
3 months	92	43%
6 months	22	10%
12 months	34	16%
More than 12 months	66	31%
**Following surgery, to who do you refer your patients?**		
Oncologist/radiation	169	79%
Respiratory physician	111	52%
General practitioner	88	41%
Physiotherapist	45	21%
Nurse specialist	22	10%
**Have you adopted a remote patient follow-up pathway?**		
Yes	60	28%
Partially	77	36%
No	77	36%
**Your remote patient follow-up is through which means?**		
Telephone	158	74%
Other	28	13%
Video conference	17	8%
Mobile application	11	5%

### Patients’ survey

The participants answered a similar but slightly modified version of the survey to reflect a similar intent behind the questions being asked but catered to the patient experience (**Table 2**).

**Table 2.** Patient Survey

Questions/answers	Total number	%
**Were you guided through the pathway by a pulmonary nurse?**		
Yes	71	31%
Partially	32	14%
No	69	30%
Don’t know	56	25%
**My preoperative path was well planned, and I had full clarity about the sequence of the different steps.**		
Fully agree	93	40%
Agree	77	33%
Neutral	33	14%
Disagree	18	8%
Strongly disagree	9	4%
**I received all of the expected information about the surgical procedure, including possible complications.**		
Fully agree	104	45%
Agree	78	34%
Neutral	28	12%
Disagree	14	6%
Strongly disagree	7	3%
**I understood all of the expected information about the surgical procedure, including possible complications.**		
Fully agree	104	45%
Agree	76	33%
Neutral	28	12%
Disagree	16	7%
Strongly disagree	7	3%
**In my opinion, I think the surgery went…**		
Better than expected	92	40%
As expected	109	48%
Worse than expected	29	13%
**At the first post-surgery appointment, I received all information about the actions required to plan follow-up for my cancer and additional treatment if needed.**		
Fully agree	65	28%
Agree	78	34%
Neutral	48	21%
Disagree	25	11%
Strongly disagree	14	6%
**At the first post-surgery appointment, I understood all information about the actions required to plan follow-up for my cancer and additional treatment if needed.**		
Fully agree	71	31%
Agree	71	31%
Neutral	50	22%
Disagree	26	11%
Strongly disagree	12	5%
**After the first surgical follow-up, who else was involved in your recovery care?**		
Oncologist/radiation	92	40%
Specialized doctor	92	40%
Nurse specialist	74	32%
General practitioner	55	24%
Surgeon	53	23%
Physiotherapist	30	13%
Unsure	23	10%
**If you were offered the option to monitor your recovery with a phone application that could record data, connect with your surgeon and give you advice on how to maximize your recovery, how comfortable would you feel?**		
Very comfortable	88	38%
Somewhat comfortable	44	19%
Comfortable	62	27%
Somewhat uncomfortable	42	18%
Uncomfortable	16	7%
Not applicable	16	7%

**Figure 3.**
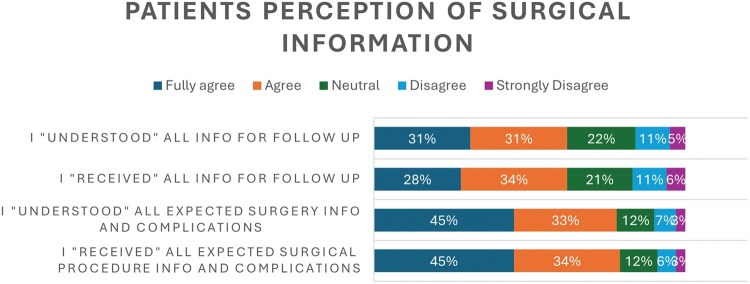
Patients’ Perception of Surgical Information, a Collection of 230 Responses per Each Agree Statements

**Figure 4.**
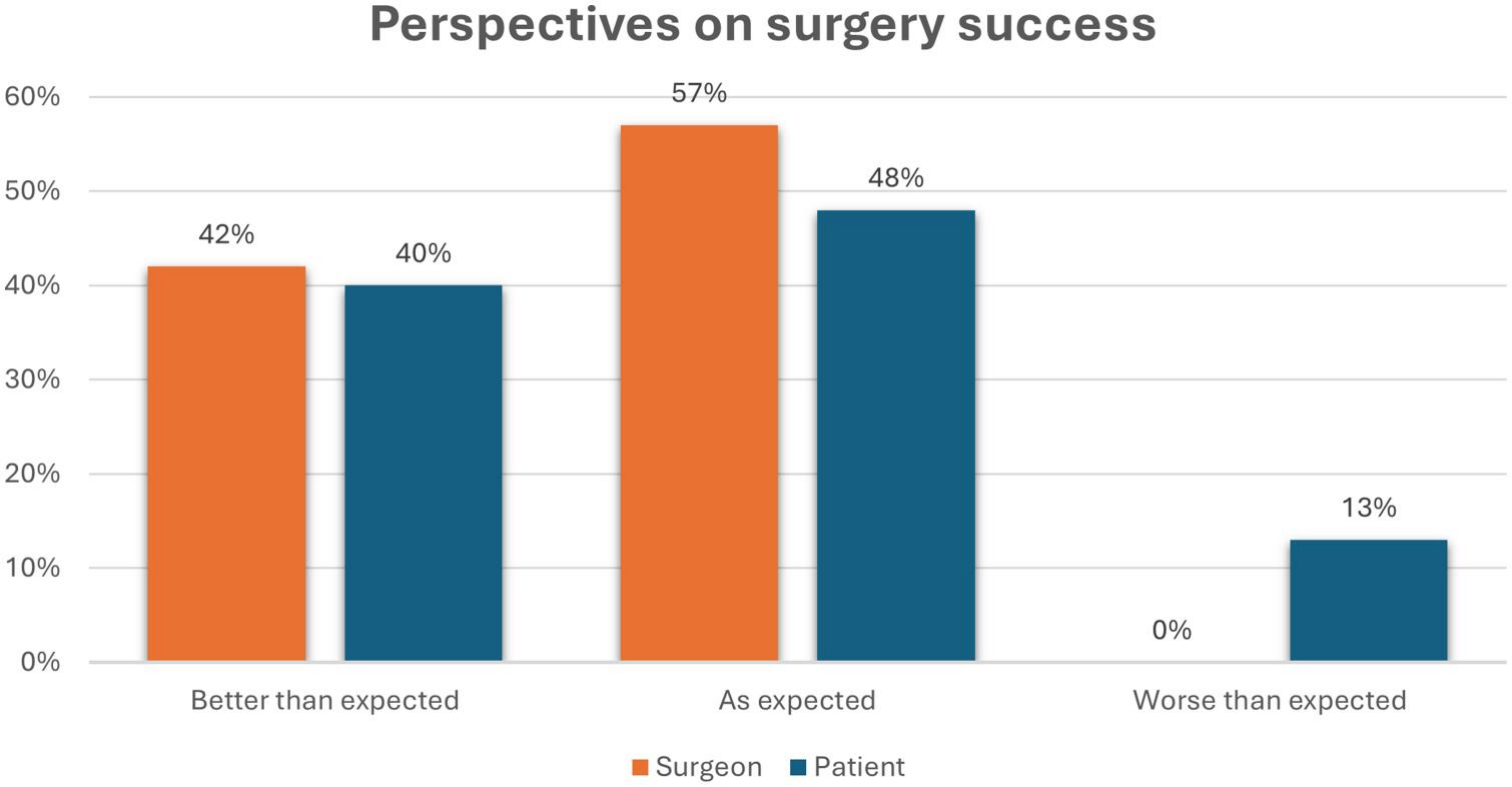
Perspectives of Surgeon and Patient Based on the Question “How Do You Think the Surgery Went?”

Only approximately one-third were guided completely through the treatment pathway by a specialized pulmonary nurse. However, most felt their perioperative path was well planned with clear sequential steps (73% fully agree and agree). Additionally, most participants felt that they received (79% fully agree and agree) and understood (78% fully agree and agree) all the information about the surgery and its possible complications (**Figure 3**). A large portion of them felt that the surgery went as expected (48%) with many responding that it went better than expected (40%) but 13% felt the surgery went worse than expected. Of note, none of the surgeons felt the surgery went worse than expected, as shown in **Figure 4**. Using a chi-square test, we found a highly significant difference (*P* < .0001), indicating a marked disparity in perception between surgeons and patients.

Only 40% of patients reported that a radiation/medical oncologist or a specialized physician was involved in their care after surgery with only 23% responding that a general practitioner was involved in their care. Most patients felt that if given the option for remote follow-up via an app on a phone they would be comfortable to very comfortable (84%).

## DISCUSSION

The results of this multi-country European survey reveal significant differences in the perioperative perceptions and experiences of people undergoing thoracic surgery and underscore the existing communication gap between patients and surgeons. The findings highlight critical areas where improvements are needed to foster a real and informed patient-surgeon shared-decision-making.

Our study indicates that while a majority of surgeons report successfully adopting ERAS protocols (80%), only a fraction of patients experienced similar levels of specialized nursing support (approximately one-third guided by a pulmonary nurse). This discrepancy suggests a potential deficiency in the implementation of comprehensive patient-centred care models in clinical practice. Since ERAS is designed to enhance recovery through collaborative processes and evidence-based interventions, ensuring patients receive adequate support throughout their surgical journey is of high importance. This reinforces existing literature on overall implementation deficits found in other studies citing that even with optimal conditions, only a 70%-75% implementation rate could be achieved.^12^^,^^13^ The proposed solutions that were made to improve implementation were as follows: the need to simplify the complexity of some of the protocols, the need for more effective teamwork and leadership, and proper dissemination of the practice within the healthcare field.^14–16^

An interesting inflection point of communication that could potentially result in downstream miscommunication events occurs in the preoperative question posed on the survey that states: “which possible complications are fully communicated to patients?” While most of the patients agreed that they received and understood all the expected information about their procedure and potential complications, the surgeons reported to communicate before and after surgery in quite different ways. For example, 92% of surgeons communicated fully about prolonged air leak, while only 77% communicated fully about infection, with decreasing percentages on mortality (61%), respiratory failure (54%) and pulmonary embolism (44%). We can postulate that if a large percentage of patients are not aware or prepared for a potential complication, then a known adverse event of a procedure can blindside a patient and caregiver family, underscoring deficiencies in care or resources.

The reported information mismatch potentiates the notion that the patient might not have had the necessary support to accurately go through their surgical experiences. This is a clear and critical gap in communication that we have identified in our survey. The reason behind less emphasis on the other complications remains unclear in our study and warrants further investigation. Nonetheless, this trend is also observed in other specialities, suggesting a broader pattern in patient priorities. Pass et al studied patient and physician interactions with 178 breast cancer patients when discussing their surgical treatment options and found that patient’s heard benefits discussed much more than risks and side effects (81% vs 58%, *P* < .001).^17^ We also see that specialities outside of surgery can cause negative outcomes as well. For example, in chronic pain control studies, deficiencies in communication have led to greater pain intensity, poorer pain relief, and poorer ratings of mood and wellbeing.^18^^,^^19^ We can also see how effective communication benefits patient outcomes as well. A systematic review was performed with the “teach-back method,” which is a technique used in patient communication where the patient is required to explain back to the physician their treatment, risks, and benefits after being informed by the physician. They found that the odds of overall 30-day readmission for discharge education and usual care were significantly less when the teach-back method was employed and the patient was able to clearly explain, therefore, comprehend all of the health-related information.^20^

The study also highlights that many patients experience uncertainty regarding postoperative care and follow-up, with a significant percentage expressing neutrality or even disagreeing that they received and understood their follow-up care instructions (21%-22% neutral, 11% disagree, 5%-6% strongly disagree) seen in **Figure 3**. This uncertainty is concerning, as postoperative clarity is critical for effective recovery and patient satisfaction. A promising avenue to address this issue is through technology. As technology advances, the integration of mobile follow-up is imminent and a potential additive to the patient care journey. As noted in recent studies, integrating digital health solutions could address barriers to communication and improve patient engagement in their care pathways.^21^^,^^22^ Of course, with growing technology, there could potentially be new digital literacy-related inequalities that can also widen the gap between countries. Better support should be ensured to avoid this potential discriminatory risk when considering digital implementation in healthcare.

Interestingly, the survey also illustrates a gap in the involvement of general practitioners in postoperative care, with only 23% of patients reporting engagement with a general practitioner post-surgery. Strengthening the role of general practitioners in the postoperative phase could facilitate better continuity of care and further personalize the patient recovery experience. A nationwide American study looked at data of over 350,000 patients and found that the rates of readmission after emergency general surgery declined significantly when there was follow-up from a general practitioner within 30 days of discharge.^23^ A cohort study performed by Brooke et al showed that 30-day readmission was lower in patients undergoing procedures with a high risk of complications and complicated hospital courses if they were followed up within 30 days with a general practitioner.^24^ This transition from longer hospitalization to a shorter length of stay but recurrent postoperative to outpatient visits is critical for patients with many complex steps needing to occur, such as, reconciling medications, communication between highly specialized physicians, organizing caregivers and much more. Of course, there are multiple different types of postoperative general care throughout Europe, including system with general practitioner involvement in the surgical follow-ups, or structured multi-disciplinary team discussions for all the suspected and surgically treated lung cancer cases. Regardless of the follow-up models, what is still of paramount importance is that someone is guiding patients and their families through the complicated process. Deficiencies in communication within the postoperative period are estimated to affect the quality of care and outcomes in more than 25% of discharged patients.^25^ Since there are many models that are utilized throughout Europe for postoperative care, future-focused studies can compare models within each region, culture, or country to identify which will provide the most optimal course.

This is a retrospective survey has some potential limitations that may have affected our results. For example, it may be true that surgeons who participate in extracurricular surveys may be more likely to implement or have already implemented ERAS-based patient-centred care. There is also an inherent recall bias to patient care, but even if a patient or surgeon may not remember precise details about their care, their perception is still not reported.

Additionally, even though our responses are represented among many different European countries, we could not determine specific countries of origin on the patient side due to patient privacy restrictions, so it is difficult to truly determine that there are not cultural factors biasing our results and to potentially compare their opinion directly with their country-specific healthcare professionals. Lastly, since we utilized social media to recruit participants who underwent thoracic surgery, we were unable to determine how many individuals viewed the survey and chose to respond, resulting in no response rate. However, the use of social media allowed us to reach a larger pool of participants, ultimately enhancing and strengthening our study.

## CONCLUSION

In conclusion, this study sheds light on critical gaps in the communication and perception of surgical care between patients and surgeons, emphasizing the need for shared decision-making and enhanced support mechanisms throughout the perioperative journey. As person-centred care continues to gain ground, healthcare systems must prioritize bridging these gaps to foster better outcomes and satisfaction. Future research should aim to explore interventions that effectively integrate PROMs into clinical practice, ensuring that surgeries not only meet clinical metrics but also align closely with patients’ needs and preferences. Additionally, strategies to educate both patients and healthcare providers on the value of collaborative care will be necessary for developing a more holistic perioperative experience.

## Supplementary Material

ivaf228_Supplementary_Data

## Data Availability

The data that support the findings of this study are available on reasonable request. The data are not openly available to pre-serve the privacy of the participants included in this study.
