# Supplementary material for: Patient-Surgeon Communication in Thoracic Surgery: Insights From a European Multi-Country Survey on the Perioperative Experience
Source: Interdiscip Cardiovasc Thorac Surg. 2025 Sep 25;40(10):ivaf228. doi: 10.1093/icvts/ivaf228 (PMC12534904; doi:10.1093/icvts/ivaf228)
Supplement: ivaf228_Supplementary_Data [file ivaf228_supplementary_data.pdf]

Questions for the patients

1. Were you guided through the care pathway by a pulmonary nurse (a specialized nurse focused on the care and treatment of patients with lung cancer or respiratory conditions)?
2. How much do you agree with the following statement? "My preoperative (before surgery) path was well planned and I had full clarity about the sequence of the different steps."
3. How much do you agree with the following statment? "I received all of the expected information about the surgical procedure, including possible complications."
4. How much do you agree with the following statment? "I understood all of the expected information about the surgical procedure, including possible complications."
5. In my opinion, how did the surgery go? How much do you agree with the following statement? "At the first post-surgery appointment, I received all information about the actions required to plan follow-up for my cancer and any additional treatment if needed."
6. How much do you agree with the following statement?
7. "At the first post-surgery appointment, I understood all information about the actions required to plan follow-up for my cancer and any additional treatment if needed."
8. Who was involved in the recovery of your care?
9. If you were offered the option to monitor your recovery with a phone application that could record data, connect with your surgeon and give you advice on how to maximize your recovery, how comfortable would you feel?

Questions for the Surgeons

1. Do you have specialized pulmonary nurses (nurse focused on the care and treatment of patients with lung cancer or respiratory conditions) in your department?
2. How successful have you been at adopting Enhanced Recovery After Surgery (ERAS) in your department?
3. Pre-operatively, which possible complications are fully communicated to patients?
4. Who communicates with the patient/family after the surgery?
5. On average, your patients feel the surgery went...
6. On average, for how long do you see your patients in clinic after discharge?
7. Following surgery, who do you refer your patients to?
8. Have you adopted a remote patient follow-up pathway?
9. Your remote patient follow-up is through which means?
